# News and Miscellany

**Published:** 1905-03

**Authors:** 


### News and Miscellany.

No other medical legislation is likely to be enacted this session.

---

Don't fail, doctor, to be at Houston next April. It will be the "rousingest" meeting we ever had, and we will have the best time.

---

Library and Instruments for Sale.—The widow of the late Dr. James Bartlett, of San Antonio, wants to sell the doctor's medical books and instruments. List, with prices, on request. Address her, care San Antonio Drug Co., San Antonio, Texas.

---

"If We Could Only Advertise in two medical journals, The Texas Medical Journal would be one of them.

"Sharpe & Dohme, New York."

(In renewing for the twenty-first year).

---

A Bill for a State sanitarium for consumptives to cost $250,-

000 has been introduced in the House by Wilmeth. It will hardly get a hearing, I fear.

THE ANATOMICAL BILL, it is thought will pass. Up today (10th.)

